# HPB International Correspondence

**DOI:** 10.1155/1995/56486

**Published:** 1995

**Authors:** René Adam, Henri Bismuth

**Affiliations:** Hepatobiliary Surgery Center Hopital Paul Brousse 12 avenue Paul Vaillant Couturier Villejuif Cedex 94804 France


3. Molleke, K., Stahl, E., Bretzke, G. (1979) Morphologische und Klinische Leberbefunde nach einaahme oraler Kontrazeptiva. *Z. Ges. Inn Med.*, **34**, 79–81.
4. Borgonovo, G., Bourokba, N., Vons, C., Mariette, D., Smadja, C., Franco, D. (1993) Prediction of blood transfusion during liver resection. *HPB Surgery*, **6 (suppl)** 13.
5. Bismuth, H., Castaing, D., Garden, O. J. (1989) Major hepatic resection under total vascular exclusion. *Ann. Surg.*, **210**, 13–19.
6. Pateron, D., Babany, G., Belghiti, J., et al. (1991) Giant hemangioma of the liver with pain, fever and abnormal liver tests. Report of two cases. *Dig. Dis. Sci.*, **36**, 524–527.
7. Trastek, V. F., Van Heerden, J. A., Sheedy, P. F., Adson, M. A. (1983) Cavernous hemangioma of the liver: resect or observe? *Am. J. Surg.*, **145**, 49–53.
8. Sejourne, P., Poirier, A., Meakins, J. L., Chamier, F., Smadja, C., Grange, D., Franco, D. (1989). Effect of haemodilution on transfusion requirements in liver resection. *Lancet*, **2**, 1380–1282.

Professor Dominique Franco, MD

Hôpital Antoine Béclère

157 rue de la Porte de Trivaux

92141 Clamart Cedex

France

### HPB INTERNATIONAL CORRESPONDENCE

*Editorial: Partial portacaval shunt: Narrow diameter H-graft by J. A. Myburgh. Ref: HPB Surgery; 8(1): 57–59*

### COMMENT BY R. ADAM AND H. BISMUTH

Professor Myburgh's commentary makes several interesting observations about our paper. However, we would like to discuss his comment that by extrapolation of our results it can be assumed that 68% of our patients with calibrated porta caval shunts lost hepatopetal portal perfusion.

- In patients with 10 mm grafts, our data showed that hepatopetal flow was maintained at 1 year in 7 of 10 patients (70%) who underwent arteriography. Logically if these findings are extrapolated to our series of 19 patients with 10 mm grafts one would expect to find hepatopetal flow in 13 patients.
- In patients with 12 mm grafts, as Professor Myburgh states, the earlier work of Sarfeh and his colleagues[1,2] using a combination of fluoroscopy and selective angiography demonstrated that only 1 of their 12 patients receiving a 12-14 mm graft maintained pro-grade portal flow at one week after surgery. Therefore, we may assume that none of the four patients with 12 mm grafts of our series have maintained prograde portal flow.
- In patients with 8 mm grafts, as nine of the 11 patients of Sarfeh's series maintain prograde portal flow, we may assume that our two patients with 8 mm graft would have retained prograde flow.

Overall consideration of our 25 patients gives a total of 15 patients (60%) who retained prograde flow and not 32% as suggested by Professor Myburgh. Nevertheless we would agree with him that there is no absolute correlation between the occurrence of encephalopathy and maintained portal venous perfusion.

In this respect, the work of Sarfeh (2) revealed a correlation between the occurence of encephalopathy and the presence of reversed flow (35% encephalopathy in those with reversed portal flow compared with 9% in patients with prograde portal flow $p = 0.02$) but other workers have reported that factors other than straightforward preservation of pro-grade portal flow are involved in the development of post operative ancephalopathy-for example, augmentation of hepatic arterial perfusion of the liver after partial decompression[3] and maintenance of mesenteric venous hypertension limiting the absorption of nitrogenous compounds[4].

We are currently evaluating the long term results of surgery in these patients and in further patients with cirrhosis who have undergone the calibrated partial portocaval shunt. The results in our series which now stands at 43 patients tends to support the earlier work. Namely, the operative mortality rate is low (2%), the rate of variceal re-bleeding remains low (2%) and acute encephalopathy was seen in 4 patients (9%) and chronic encephalopathy in 2(5%). The actuarial survival at five years is 82%. These results augment Professor

Myburgh's concluding remarks that the small diameter ringed PTFE interposition prosthetic graft has established a place in the therapeutic armamentarium for portal hypertension.

### REFERENCES

1. Sarfeh, I. J., Rypins, E. B., Fardi, M. et al. (1984). Clinical implication of portal hemodynamics after small-diameter portocaval H graft. *Surgery*, **96**, 223–229.
2. Sarfeh, I. J., Rypins, E. B. and Mason, G. R. (1986) A systematic appraisal of portacaval H-diamters. *Ann. Surg.*, **204**, 356–363.
3. Johansen, K. (1993) Basis for beneficial effect of partial portal decompression. *Surgery*, **114**, 129–130 (letter).
4. Rikkers, L. F. (1983) Portal hemodynamics intestinal absorbtion and post shunt encephalopathy. *Surgery*, **94**, 126–133.

René Adam, MD, PhD
Henri Bismuth MD, FACS
Hepatobiliary Surgery Center
Hopital Paul Brousse
12 avenue Paul Vaillant Couturier
94804 Villejuif Cedex
France

### REPLY BY J. A. MYBURGH

Henri Bismuth's calculations are correct; namely retention of prograde flow in 60%. My figure of 32% was the result of a silly miscalculation which slipped through. I would suggest that I acknowledge this error as follows:
"Professor Bismuth's calculation is quite correct. I apologise for the regrettable miscalculation in my commentary of an excellent contribution".

J. A. Myburgh
Emeritus Professor of Surgery
University of the Witwatersrand
York Road
Parktown
Johannesburg 2193
South Africa

